# Risk factors associated with progression of diabetic retinopathy in eyes treated with panretinal photocoagulation

**DOI:** 10.1038/s41598-021-93384-5

**Published:** 2021-07-05

**Authors:** Sung Uk Baek, Min Seon Park, Bum-Joo Cho, In Won Park, Soonil Kwon

**Affiliations:** Department of Ophthalmology, Hallym University Sacred Heart Hospital, Hallym University College of Medicine, 22, Gwanpyeong‑ro 170beon‑gil, Dongan-gu, Anyang, 14068 Republic of Korea

**Keywords:** Diseases, Medical research

## Abstract

Uncontrolled diabetes has been associated with progression of diabetic retinopathy (DR) in several studies. Therefore, we aimed to investigate systemic and ophthalmic factors related to worsening of DR even after completion of panretinal photocoagulation (PRP). We retrospectively reviewed DR patients who had completed PRP in at least one eye with a 3-year follow-up. A total of 243 eyes of 243 subjects (mean age 52.6 ± 11.6 years) were enrolled. Among them, 52 patients (21.4%) showed progression of DR after PRP (progression group), and the other 191 (78.6%) patients had stable DR (non-progression group). The progression group had higher proportion of proliferative DR (*P* = 0.019); lower baseline visual acuity (*P* < 0.001); and higher platelet count (*P* = 0.048), hemoglobin (*P* = 0.044), and hematocrit, (*P* = 0.042) than the non-progression group. In the multivariate logistic regression analysis for progression of DR, baseline visual acuity (HR: 0.053, *P* < 0.001) and platelet count (HR: 1.215, *P* = 0.031) were identified as risk factors for progression. Consequently, we propose that patients with low visual acuity or high platelet count are more likely to have progressive DR despite PRP and require careful observation. Also, the evaluation of hemorheological factors including platelet counts before PRP can be considered useful in predicting the prognosis of DR.

## Introduction

Diabetic retinopathy (DR) is the leading cause of decreased vision and blindness among working-age adults in most developed countries^1–3^. Chronic hyperglycemia in diabetes mellitus (DM) causes impairment of capillaries, resulting in retinal ischemia and increase of vascular permeability. DR progresses from nonproliferative diabetic retinopathy (NPDR) to proliferative diabetic retinopathy (PDR), a stage that can cause blindness due to the formation of retinal neovascularization, resulting in vitreous hemorrhage or tractional retinal detachment^4,5^.

Panretinal photocoagulation (PRP) has been the main treatment to prevent severe vision loss in patients with severe NPDR or PDR according to the findings of studies on DR, and the effectiveness of the treatment was confirmed by the early treatment diabetic retinopathy study (ETDRS)^6^. PRP is usually completed in 4 or more sessions. Complete PRP has reduced the five-year risk of blindness in patients with PDR by over 90%^7^. But, several studies have reported that 45% of the eyes that were treated with PRP needed additional PRP to manage active PDR^8^. Susan et al. reported that PDR worsening occurred in 42% of those in the PDR treated with PRP group and in 34% of those in the PDR treated with ranibizumab group^8^. It was revealed that uncontrolled diabetes, diabetic nephropathy, anemia, etc., were associated with progression of PDR in several studies. However, there were a few studies that have focused on systemic factors associated with worsening of PDR after completion of PRP^8^.

Therefore, we aimed to explore systemic factors related to worsening of PDR after completion of PRP using the clinical data warehouse (CDW) system. To explore the large database of clinical information of patients with PDR, we used the CDW system of the hospital, which is an electronic data repository of patient and provider information. This is one of the largest studies on the association of systemic conditions such as comorbid diseases with PDR utilizing clinical data and laboratory test results in Asians. The findings of this study are expected to provide insight into the pathogenesis of PDR progression.

## Results

The data of 1052 subjects with DR were extracted from the CDW system. After excluding patients who did not meet the criteria, a total of 243 eyes of 243 individuals were eligible for this study. Of 243 patients, 52 (21.4%) who showed progression of DR after PRP were defined as the progression group, and the other 191 (78.6%) patients comprised the non-progression group.

### Laboratory and ophthalmic findings

Table 1 shows clinical characteristics of all participants and the comparison between the progression and non-progression group at baseline. The mean age of the total study group was 52.6 ± 11.6 years and the mean duration of DM was 15.5 ± 7.85 years, with a mean hemoglobin A1c (HbA1c) level of 8.65 ± 1.80%. Out of 243 patients, there were 50 (20.6%) patients with severe NPDR and 193 (79.4%) patients with PDR. PDR patients were significantly higher in the progression group than in the non-progression group (90.4% vs 76.4%, *P* = 0.019). In particular, the baseline visual acuity was significantly lower in the progression group (0.47 ± 0.33) than in the non-progression group (0.69 ± 0.29) (*P* < 0.001). Platelet count, hemoglobin, and hematocrit were higher in the progression group than in the non-progression group (*P* = 0.035, 0.014, 0.011, respectively). Phosphorus, uric acid and total cholesterol values were significantly different between two groups (*P* = 0.048, 0.044, 0.042, respectively). Other laboratory findings did not show any statistical significance.

**Table 1:** Comparison of clinical characteristics between progression and non-progression group.

	Total (N = 243)	Progression (N = 52)	Non-progression (N = 191)	*P* value*
Age, years	52.6 ± 11.6	50.2 ± 11.9	53.7 ± 11.4	0.325
Sex ratio, male/female	136:107	29:23	107:84	0.992
Systemic hypertension, n (%)	130 (53.5)	35 (67.3)	95 (49.7)	0.144
Ischemic heart disease, n (%)	23 (9.5)	4 (7.7)	19 (9.9)	0.815
Cerebrovascular disease, n (%)	32 (13.2)	12 (23.1)	19 (9.9)	0.122
Chronic kidney disease	71 (29.2)	18 (34.6)	53 (27.6)	0.331
Body mass index (kg/m^2^)	25.06 ± 5.23	24.87 ± 5.09	24.84 ± 4.09	0.935
Duration of DM (years)	15.5 ± 7.85	15.3 ± 8.2	15.5 ± 7.7	0.806
**Body mass index (kg/m**^**2**^**)**	24.96 ± 4.10	24.87 ± 5.10	24.85 ± 4.09	0.896
Thin (< 20 kg/m^2^) (n)	25	6 (11.5)	19 (9.9)	0.414
Normal (20 to < 25)	111	26 (50.0)	85 (44.5)	0.788
Overweight overweight (25 to < 30)	81	13 (25.0)	68 (35.6)	0.124
Obese (≥ 30)	26	7 (13.5)	19 (9.9)	0.472
Cigarette smoking status (non-smokers/ex-smokers/current smokers)	155/25/63	29/5/18	126/20/45	0.868
Duration of follow-ups (years)	5.91 ± 2.81	5.94 ± 2.86	5.90 ± 2.80	0.633
**Retinal level, no. (%)**				
Severe NPDR	50 (20.6)	5 (9.6)	45 (23.6)	
PDR	**193 (79.4)**	**47 (90.4)**	**146 (76.4)**	**0.019**
**Ophthalmic variables**				
IOP, mmHg	15.29 ± 3.94	15.86 ± 3.94	15.14 ± 3.37	0.229
Visual acuity (Snellen)	**0.66 ± 0.31**	**0.47 ± 0.33**	**0.69 ± 0.29**	** < 0.001**
Spherical equivalent, diopters	− 0.31 ± 1.71	− 0.29 ± 1.29	− 0.33 ± 1.80	0.822
Axial length, mm	23.29 ± 0.97	23.23 ± 1.05	22.23 ± 4.96	0.975
Lens status (phakic/pseudophakic)	196/47	40/12	156/35	0.876
Glaucoma accompanied, n (%)	28 (11.5)	6 (11.5 )	22 (11.5)	0.650
**Blood concentration of (103/μl)**				
Platelet count	256.6 ± 68.2	285.08 ± 77.83	248.13 ± 60.37	0.035
MPV (fL)	8.26 ± 1.06	8.29 ± 1.18	8.24 ± 0.94	0.802
PDW (%)	48.8 ± 10.5	46.8 ± 9.3	50.5 ± 11.1	0.073
Hemoglobin	**12.9 ± 2.0**	**12.28 ± 1.84**	**13.03 ± 2.02**	**0.014**
Hematocrit	**37.9 ± 5.7**	**36.22 ± 5.15**	**38.35 ± 5.75**	**0.011**
HbA1c (%)	8.65 ± 1.80	8.68 ± 1.82	8.55 ± 1.73	0.577
**Chemistry**				
Sodium (Na), mmol/L	140.1 ± 19.6	138.69 ± 3.76	140.47 ± 20.11	0.865
Potassium (K), mmol/L	4.61 ± 0.53	4.67 ± 0.51	4.59 ± 0.53	0.418
Chloride (Cl), mmol/L	101.7 ± 4.3	101.77 ± 4.91	101.71 ± 4.16	0.904
TCO_2_, mmol/L	25.98 ± 3.57	27.14 ± 10.51	26.04 ± 3.54	0.251
Calcium, total (mg/dL)	9.34 ± 0.58	9.38 ± 0.68	9.33 ± 0.56	0.180
Phosphorus (mg/dL)	3.7 ± 0.7	3.89 ± 0.84	3.64 ± 0.68	0.048
Uric acid (mg/dL)	5.5 ± 1.8	5.94 ± 1.45	5.37 ± 1.88	0.044
Albumin (mg/dL)	4.2 ± 0.4	4.15 ± 0.55	4.25 ± 0.42	0.747
Protein, total (g/dL)	7.1 ± 0.6	7.13 ± 0.83	7.13 ± 0.58	0.918
ALT (mg/dL)	22.1 ± 15.5	19.17 ± 9.95	23.18 ± 16.61	0.057
AST (mg/dL)	24.5 ± 20.4	21.77 ± 10.82	25.19 ± 22.22	0.159
Cr eGFR (eGFR, mean (SD), mL/min/1.73 m^2^)	73.1 ± 30.0	68.49 ± 31.92	74.33 ± 29.44	0.460
BUN (mg/dL)	21.6 ± 11.6	23.90 ± 14.11	20.93 ± 10.76	0.185
Creatinine (mg/dL)	1.4 ± 1.5	1.62 ± 1.85	1.32 ± 1.41	0.210
Protein (urine) (grade 1 ~ 4)	0.8 ± 1.1	0.99 ± 1.20	0.70 ± 1.08	0.900
**Lipid profile**				
Total cholesterol (mg/dL)	**180.9 ± 51.3**	**196.72 ± 63.90**	**176.60 ± 46.67**	**0.042**
Triglycerides (mg/dL)	152.8 ± 105.7	166.28 ± 98.31	143.06 ± 80.72	0.124
LDL cholesterol (mg/dL)	99.5 ± 41.5	103.59 ± 55.84	98.46 ± 37.25	0.518
HDL cholesterol (mg/dL)	46.4 ± 12.8	44.16 ± 13.05	46.92 ± 12.80	0.329
**Blood coagulation test**				
PT (prothrombin time) (s)	13.7 ± 3.3	14.38 ± 4.96	13.43 ± 2.60	0.202
aPTT (s)	36.5 ± 12.8	35.22 ± 7.36	36.21 ± 10.72	0.456

Bolded values represent significance (*P* < 0.05).

DM = diabetic mellitus; NPDR = nonproliferative diabetic retinopathy; PDR = proliferative diabetic retinopathy; IOP = intraocular pressure; MPV = mean platelet volume; PDW = platelet distribution width; TCO2 = total carbon dioxide; Cr eGFR = creatine estimated-glomerular filtration rate ALT = alanine aminotransferase; AST = aspartate aminotransferase; BUN = blood urea nitrogen; LDL = low-density lipoprotein; HDL = high-density lipoprotein; PT = prothrombin time; aPTT = activated partial thromboplastin time.

*P values are derived from comparison between Progression and Non-progression group.

Of the total number of patients, 39 (16.0%) underwent vitrectomy. In the non-progression group, five vitrectomies (2.6%) were done to treat refractory diabetic macular edema and the long-lasting vitreous hemorrhage. The number of patients who had intravitreal injections of bevacizumab, ranibizumab, and triamcinolone acetonide were 40 (16.5%), 3 (1.2%), and 11 (4.5%), respectively.

### Risk factors for progression of DR

The systemic and ocular risk factors for progression of DR were analyzed using logistic regression analysis as shown in Table 2. In the univariate analysis, severity of DR, visual acuity at presentation, platelet count, hemoglobin, hematocrit, phosphorus, uric acid, and total cholesterol were associated with worsening of DR after completion of PRP. However, after adjusting for systemic and ophthalmic factors, only visual acuity (HR: 0.053, CI: 0.012–0.232, *P* < 0.001) and platelet count (HR: 1.215, CI: 1.097–1.309, *P* = 0.031) were identified as risk factors for DR progression in multivariate analysis.

**Table 2:** Univariate and multivariate logistic regression analysis model data for progression of DR.

	Univariate			Multivariate		
	Hazard ratio	95% confidence interval	*P* value	Hazard ratio	95% confidence interval	*P* value
Age, years	0.986	0.961–1.013	0.311			
Sex ratio, male/female	0.997	0.537–1.850	0.992			
Systemic hypertension, n (%)	1.475	0.859–2.533	0.159			
Ischemic heart disease, n (%)	0.981	0.924–1.041	0.528			
Cerebrovascular disease, n (%)	0.919	0.559–2.604	0.233			
**Body mass index (kg/m**^**2**^**)**	0.995	0.987–1.003	0.813			
Duration of DM, years	0.995	0.956–1.035	0.797			
Body mass index (kg/m^2^)	0.997	0.926–1.074	0.934			
Thin (< 20 kg/m^2^) (n)	1.009	0.966–1.054	0.686			
Normal (20 to < 25)	1.195	0.724–2.298	0.588			
Overweight (25 to < 30)	0.763	0.606–1.069	0.102			
Obese (≥ 30)	1.009	0.966–1.054	0.686			
Cigarette smoking status (non-smokers/ex-smokers/current smokers)	0.957	0.506–1.809	0.892			
Severity of DR, PDR	**3.051**	**1.164–7.996**	**0.023**	1.595	0.597–4.259	0.351
**Ophthalmic variables**						
IOP, mmHg	1.064	0.970–1.166	0.190			
Visual acuity at presentation (Snellen)	**0.043**	**0.012–0.159**	**0.000**	**0.053**	**0.012–0.232**	**0.000**
Spherical equivalent, diopters	1.020	0.832–1.249	0.851			
Axial length, mm	1.008	0.627–1.619	0.975			
Lens status (phakic/pseudophakic)	0.938	0.417–2.106	0.976			
Glaucoma accompanied	1.237	0.495–3.093	0.649			
Chronic kidney disease	1.385	0.720–2.665	0.330			
**Blood concentration of**						
Platelet count	**1.244**	**1.101–1.308**	**0.022**	**1.215**	**1.097–1.309**	**0.031**
MPV (fL)	1.181	0.790–1.767	0.417			
PDW (%)	0.960	0.920–1.002	0.063			
Hemoglobin	**0.835**	**0.716–0.974**	**0.022**	1.307	0.615–2.778	0.486
Hematocrit	**0.935**	**0.884–0.988**	**0.018**	0.862	0.660–1.126	0.277
HbA1c (%)	0.952	0.798–1.136	0.586			
**Chemistry**						
Sodium (Na)	1.001	0.989–1.013	0.927			
Potassium (K)	0.986	0.916–1.061	0.707			
Chloride (Cl)	1.004	0.935–1.079	0.903			
TCO_2_	1.027	0.978–1.078	0.291			
Calcium, total	1.298	0.864–1.949	0.209			
Phosphorus	**1.603**	**1.056–2.434**	**0.027**	1.305	0.769–2.215	0.324
Uric acid	**1.181**	**1.002–1.393**	**0.047**	1.082	0.885–1.323	0.445
Albumin	0.935	0.593–1.474	0.773			
Protein, total	1.031	0.639–1.665	0.899			
ALT (mg/dL)	0.980	0.955–1.007	0.140			
AST (mg/dL)	0.988	0.963–1.013	0.330	1.079	0.976–1.193	0.137
Cr eGFR (mL/min/1.73 m^2^)	0.996	0.987–1.006	0.455			
BUN (mg/dL)	1.016	0.992–1.041	0.190			
Creatinine (mg/dL)	1.118	0.936–1.336	0.217			
Protein (urine) (grade 1 ~ 4)	1.277	0.948–1.588	0.120			
**Lipid profile**						
Total cholesterol (mg/dL)	**1.007**	**1.001–1.013**	**0.017**	1.006	0.999–1.014	0.081
Triglycerides (mg/dL)	0.130	0.999–1.007	0.130			
LDL cholesterol (mg/dL)	1.003	0.994–1.012	0.516			
HDL cholesterol (mg/dL)	0.985	0.956–1.015	0.332			
**Blood coagulation test**						
PT (prothrombin time)	1.073	0.986–1.167	0.103			
aPTT	0.987	0.945–1.030	0.540			

Logistic regression analysis for systemic and ocular parameters in Progression group, using the Non-progression group as a reference.

Bolded values represent significant, *P* < 0.05.

### Diagnostic validity of DR progression

**Figure 1:**
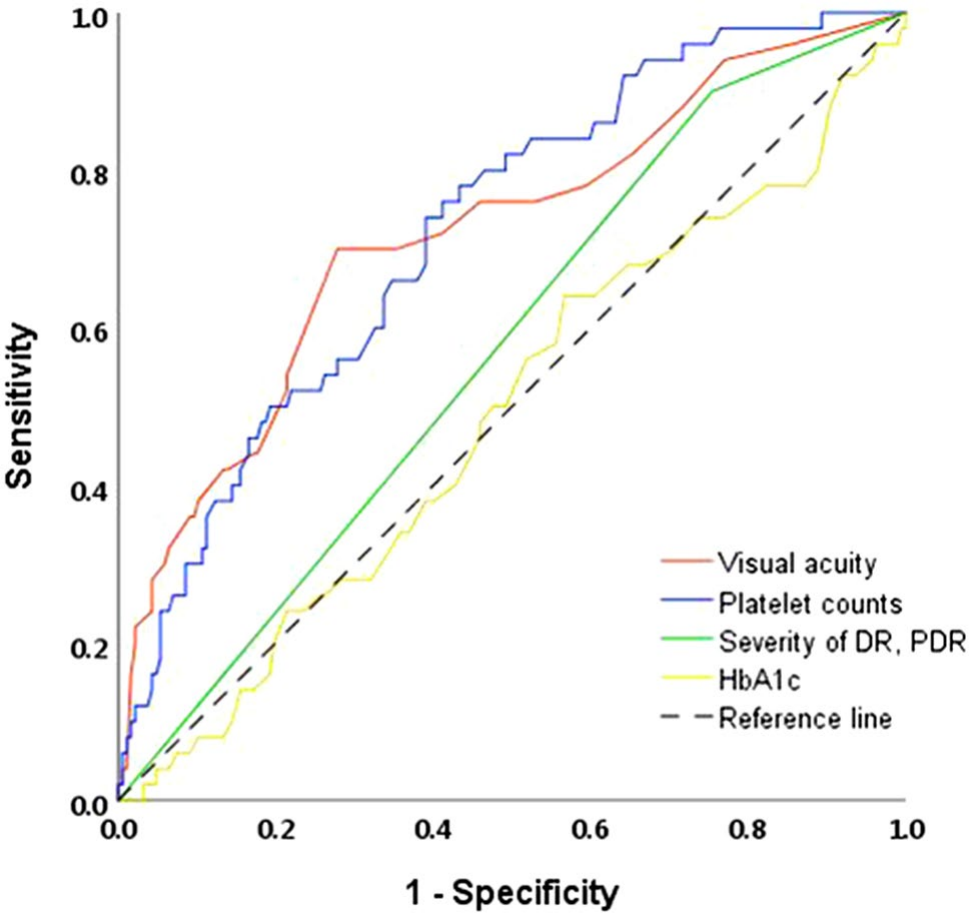
The validation of diagnostic performance between visual acuity and platelet counts. The area under the receiver operating characteristic curve (AUROC) of the visual acuity was 0.728 (*P* < 0.001, CI 0.645–0.811) and platelet counts was 0.722 (*P* < 0.001, CI 0.648–0.796). The cut off point of a high likelihood of DR progression was visual acuity, 0.505 (sensitivity: 71.2; specificity: 71.7) and platelet counts, 253 (× 103/uL) (sensitivity: 75.0; specificity: 61.2).

To validate the diagnostic performance of DR progression, the area under the receiver operating characteristic curve (AUROC) of visual acuity and platelet count were derived and then compared with severity of DR and HbA1c, known as representative risk factors for DR progression (Fig. 1). The AUROC of the visual acuity was 0.728 (*P* < 0.001, CI 0.645–0.811) and platelet count was 0.722 (*P* < 0.001, CI 0.648–0.796). However, the severity of DR and HbA1c level were identified as AUROC 0.573 (*P* = 0.113, CI 0.489–0.657) and 0.489 (*P* = 0.815, CI 0.399–0.580), respectively. Then, the cut-off point of visual acuity and platelet counts was calculated by considering the balance between sensitivity and specificity for the ability to distinguish the progression of DR. The cut-off point was visual acuity, 0.505 (sensitivity: 71.2%; specificity: 71.7%); and platelet counts, 253 (× 10^3^/µL) (sensitivity: 75.0%; specificity: 61.2%).

### Cumulative probability of DR progression

**Figure 2:**
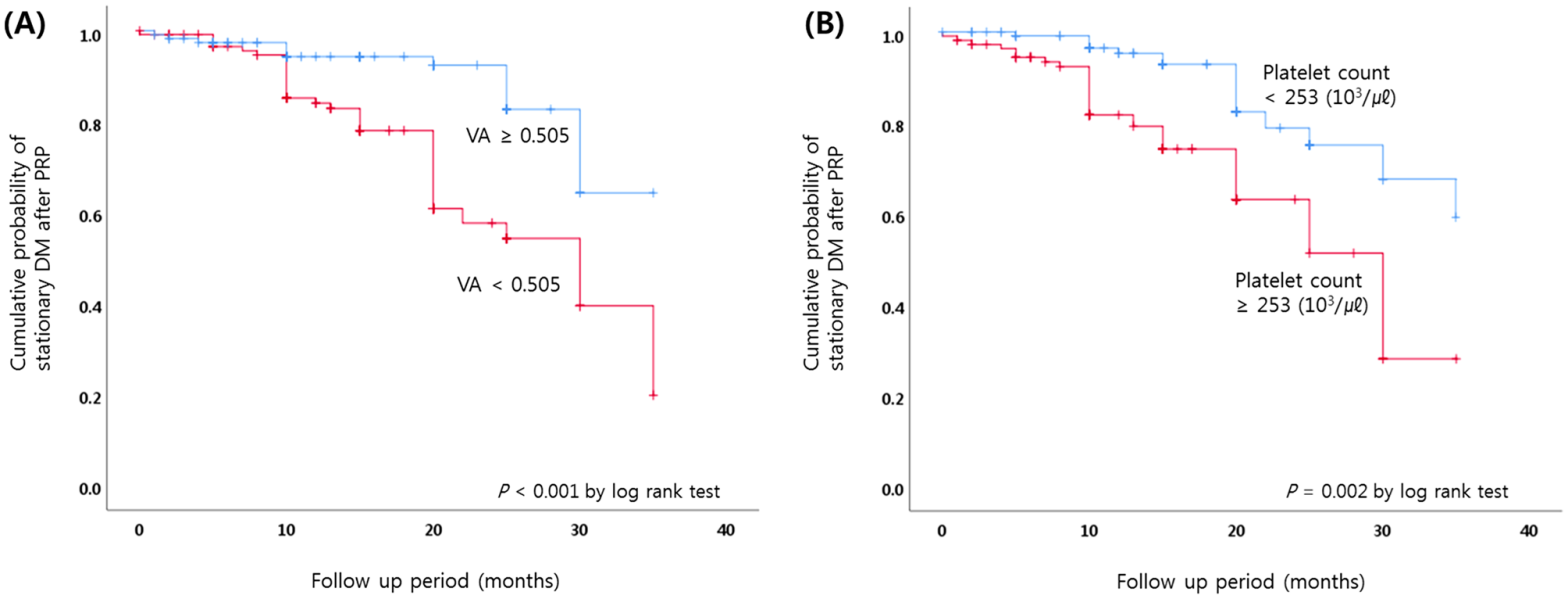
Kaplan–Meier survival analysis of stationary DR after PRP. The subgroups were stratified as (**A**) visual acuity (VA) < 0.505 or ≥ 0.505 and (**B**) platelet count < 253 or ≥ 253 (103/μl). Kaplan–Meier survival analysis was used to compare the cumulative probability of DR progression between the groups as stratified by the level of VA and platelet count. The VA and platelet count were classified into two groups based on the mean values for the total subjects.

Kaplan–Meier survival analysis was used to compare the cumulative probability of DR progression using the derived cut off point (Fig. 2). The subgroups were stratified as (A) visual acuity < 0.505 or ≥ 0.505 and (B) platelet count < 253 or ≥ 253 (10^3^/µL) (Fig. 2). Lower visual acuity (< 0.505) and higher platelet count (≥ 253 × 10^3^/µL) showed greater cumulative probabilities of DR progression (*P* < 0.001 and 0.002 by log rank test, respectively).

## Discussion

This study investigated the predictive markers for DR progression in patients after completion of PRP. A data warehouse was used to analyze a wide range of ophthalmic and laboratory variables in a large patient population. After adjusting for various confounding factors, lower visual acuity at the start of PRP among ophthalmic parameters and higher platelet count among laboratory parameters were found to be predictive of the high probability of DR progression after PRP.

The present study showed that DR significantly progressed after PRP when the initial visual acuity was low. While the action mechanism of the PRP is unknown, it is assumed that the PRP reduces metabolic demand and promotes diffusion of oxygen from the choroid to the retina^9^. However, in cases where the macular ischemic condition presenting as low visual acuity is severe, diffusion of oxygen may remain insufficient despite PRP, and DR could actually worsen. In particular, this finding in line with the result of a study that reported that low visual acuity is associated with larger foveal avascular zone size in patients with DR^10^. Poor visual acuity can imply macular ischemia, which is likely to be associated with a progression of DR. In addition, higher platelet count showed significant association with worsening of DR in the present study. Altered platelet morphology and function have been observed in diabetes in the form of enhanced platelet activity, which may contribute to this “prothrombotic state”^11^. Furthermore, a few studies reported that diabetic patients have higher thrombocyte count and platelet activation can lead to the generation of vascular diseases^12,13^. Thus, more attention needs to be paid to patients with higher platelet count for monitoring DR progression.

In this study, higher platelet count was identified as an associated factor worsening DR progression, whereas mean platelet volume (MPV) and platelet distribution width (PDW) did not show any significant association. In the case of PDW, similar findings were reported by another study indicating that PDW is not related to the severity of DR^14^. Meanwhile, MPV is associated with DM severity and increased value in PDR, hemorrhagic disease, or coronary ischemic disease^15–17^. There could be several possible explanations why MPV did not show any association with DR progression in this study. MPV could have decreased due to the influence of cytokines in acute inflammatory disease^18,19^. In the progression group, not a few patients with younger age and short duration of DM were included. On the other hand, patients with chronic renal failure and long-standing DM but relatively stable DR were included in the non-progression group. Due to these factors, an increase of MPV in the progression group might be only to a relatively small extent, despite progressive DR. For example, the next thing to be considered is that MPV and PDW are susceptible to by various factors^20^. Even the time difference between blood collection and testing of the sample can cause a significant change in these values. Therefore, for accurate measurement of MPV and PDW, test conditions should be uniform and strictly enforced. However, as this study retrospectively used CDW data spanning a long period of 7 years, it is difficult to determine if the laboratory values of MPV and PDW could have been affected due to various uncontrolled factors. In order to improve these shortcomings, a prospective complementary study with uniform testing conditions is needed.

DR severity is also well-known as a strong factor associated with DR progression. The ETDRS registered 3711 patients with mild-to-severe nonproliferative or early proliferative DR and randomly assigned each patient to undergo early photocoagulation or delayed treatment until high-risk proliferative retinopathy was detected. At 5 years, rates of severe vision loss were 2.6% with early treatment and 3.7% with deferred treatment^21^. Bressler et al. also reported that higher baseline levels of ETDRS retinopathy were associated with higher rates of supplemental PRP in the PRP group^8^. These are consistent with our results in the univariate analysis; however, severity of DR was not a significant factor in multivariate analysis. In addition, the validation of DR progression did not show any significance (AUROC 0.573). This could be explained by the relatively small proportion of severe NPDRs or the effect of other confounding variables. HbA1c has been known to be a useful marker to determine mean blood glucose levels^11^. However, HbA1c was not effective marker for prediction of DR progression after PRP in our study (AUROC 0.489). This finding is consistent with a report that total glycemic exposure only accounts for approximately 11% of the variation in DR progression, as shown in the Diabetes Control and Complications Trial^11^.

We hypothesized that several systemic factors might be associated with progression of DR. Anemia was one of the systemic factors considered by us to be associated with DR progression. Anemia is a decrease in hemoglobin concentration and a sign of reduced oxygen-carrying capacity. In this study, the level of hemoglobin and hematocrit were lower in the progression group than in the non-progression group. Although not identified as a significant factor in multivariate regression analyses, the aforementioned results suggest that lower hemoglobin and hematocrit levels support the pathophysiological understanding of the reduction in oxygen-carrying capacity that exacerbates tissue hypoxia^22^.

In present study, glaucoma was identified in 22 (11.5%) patients in the non-progression group and 6 (11.5%) patients in the progression group. There were 5 neovascular glaucoma (NVG), 6 normal-tension glaucoma (NTG), and 11 primary open-angle glaucoma (POAG) patients in the non-progression group and 4 NVG and 2 NTG patients in the progression group. Glaucoma is known to occur either by ischemia (ischemic theory) as well as through mechanical effect (mechanical theory). We hypothesized that the glaucomatous eye was more susceptible to the ischemic state; thus, the probability of DR progression would increase in glaucoma patients. However, contrary to our assumption, the proportion of NTG and POAG patients in the progression group was not high.

In our study, uric acid and total cholesterol showed significant association with DR progression in the univariate analysis but not in the multivariate analysis. Hyperuricemia has been found to independently predict the development of diabetes and mediate insulin resistance in both fructose-dependent and fructose-independent models of metabolic syndrome^23^. Abnormal total cholesterol level is a risk factor for occurrence of sight-threatening DR and diabetic macular edema, and higher serum triglyceride level is a risk factor for PDR in subjects with type 2 diabetes. Dyslipidemia with abnormal levels of total cholesterol, LDL, HDL, and triglyceride is associated with greater risk of incident diabetic macular edema and greater risk for progression to PDR, compared with abnormal levels of individual lipid levels^24^.

The exploratory analyses conducted in this report have some limitations. First, PRPs were performed by two different surgeons even though protocols were the same. Second, hematologic laboratory evaluation was done before PRP. Thus, it is not clear whether the patients managed DM well after PRP. Third, we are unaware of the exact causal relationship due to the retrospective nature of this study: whether patients with high platelet count developed PDR or whether patients with poorly managed DM had a high platelet count. Further studies are required to clearly elucidate the various systemic factors associated with progression of PDR after PRP. To definitively answer the question of systemic factors associated with worsening of PDR, a prospective study needs to be designed to study the relationship between DR progression and the function, activity, and count of platelets. Finally, for the accurate validation of the two predictive markers, visual acuity and platelet counts identified in this study, a separate validation set, which is distinct from the training set, is required. Ideally, using the training set, we should identify the predictive markers of DR progression. Then, using the independent validation set, we could confirm the predictive ability of DR progression via comparison with other parameters. In the future, additional validation studies taking into account these points should be supplemented.

In summary, low visual acuity and relatively higher platelet count are associated with ongoing progression of DR despite completion of PRP. Therefore, careful monitoring is required for patients with low visual acuity along with a thorough visual assessment prior to PRP. In addition, evaluation of hemorheological factors, including platelet count, before PRP is considered useful, and special clinical attention may be needed in patients with high platelet count. If complementary analyses of hemorheological factors, such as platelet count and HbA1c levels, are performed in large-scale prospective studies, they might be assessed to check if they could serve as useful biomarkers in predicting DR progression.

## Methods

This investigation is based on the Hallym DR Study, an ongoing cohort study conducted at Hallym University Medical Center (HUMC). To explore the large database of hospital-collected clinical information on patients with DR, we used the common integrated CDW system of HUMC, which is an electronic data repository of patients’ information^25^. A detailed description of the CDW system was also introduced in previous studies, with some modification^26,27^. The common CDW system of HUMC collects and stores extensive electronic medical data including medical records, laboratory results, physical measurements, diagnostic and therapeutic history, and medication history over a period of 10 years^27^.

We accessed the CDW system and investigated the medical data of patients who were diagnosed with DR and treated with PRP between January 2009 and December 2015. This study was approved by the institutional review board of HUMC, and all protocols were in accordance with the tenets of the Declaration of Helsinki. The need for informed consent was waived by the Institutional Review Boards of Hallym University Sacred Heart Hospital because of the retrospective nature of the study and the de-identification of data by the CDW system before we accessed the database.

### Study population

We first identified patients diagnosed with DR, Korean Standard Classification of Diseases (KCD) code H34.8, corresponding to the International Classification of Diseases, 9th Revision, Clinical Modification (ICD-9-CM) code 362.01 for DR during the study period. Next, to ensure the inclusion of patients who newly underwent PRP during the follow-up period, we verified the presence of a previous history of PRP by reviewing the visit data for all eligible patients, beginning from the earliest period for which medical records were provided by the CDW system (January 2009 for HUMC).

DR patients who have completed more than 4 sessions of PRP in at least one eye from 2009 to 2015 and who met these criteria were included: (1) followed up for at least 3 years after completing PRP; and (2) able to confirm the clinical information (underlying systemic comorbidities, physical measurements, and laboratory findings of blood tests and urine tests). Exclusion criteria were: ((1) a history of other retinal disease, neovascular age-related macular degeneration, retinal vein occlusion, posterior uveitis, or ischemic optic neuropathy; (2) a history of intraocular surgery other than uncomplicated cataract surgery; (3) media opacity rendering fundus reading difficult for diagnosis (significant cataract, asteroid hyalosis, or vitreous opacity); (4) a history of laser before PRP; and (5) advanced DR with complications requiring immediate surgical treatment^28,29^, such as vitreous hemorrhage or tractional retinal detachment at the first ophthalmologic visit.

### Outcome measurements, systemic variables

The data of the study subjects were investigated with regard to the systemic conditions at the time of receiving PRP. When two or more test results were available, values obtained at the date closest to the date of initiating PRP treatment were selected. Systemic diseases diagnosed before PRP treatment were defined as underlying comorbidities. Not all laboratory test results were obtainable for all the patients, and only those tests whose results were available for more than 80% of the study participants were included in the analyses.

Demographic characteristics included the patients’ sex and age at initial PRP. Systemic comorbidities were investigated using the KCD code system. We also investigated the presence of underlying disease that may affect retinal vasculature, including hypertension, ischemic heart disease (IHD), cerebrovascular disease, and chronic kidney disease. Physical measurements included height, weight, systolic blood pressure, diastolic blood pressure, and the body mass index (BMI). The laboratory protocol for DR included differential cell counts [platelet count, hemoglobin, hematocrit, etc.]; blood coagulation-related tests [activated partial thromboplastin time, and prothrombin time; lipid profile [total cholesterol, low-density lipoprotein cholesterol, high-density lipoprotein cholesterol, and triglycerides]; liver enzyme test, including alanine transaminase (ALT) and aspartate aminotransferase (AST) levels; and kidney function test, including blood urea nitrogen (BUN) and creatinine measurement.

Anthropometric measurements such as height and body weight were assessed. BMI was calculated as weight (kg) divided by height (m) squared. The BMI was categorized into 4 groups: BMI less than 20 kg/m^2^ (Thin), BMI 20 ~ 25 (Normal), BMI of 25–30 kg/m^2^ (Overweight), and more than 30 kg/m^2^ (Obese)^26,30^. Systolic blood pressure, diastolic blood pressure were measured in the right arm after a 5-min stabilization period using a standard mercury sphygmomanometer (Baumanometer; Baum, NY, USA). Further, the level of smoking was categorized as ‘‘have never smoked,’’ ‘‘previously smoked but no longer smoking,’’ or ‘‘currently smoking.’’

### Outcome measurements, ophthalmic variables

All patients underwent comprehensive ocular examinations, including best-corrected visual acuity (BCVA, Snellen chart), intraocular pressure, detailed slit-lamp biomicroscopy and dilated fundus examination after dilatation of the pupils, fundus photography, optical coherence tomography (OCT) imaging, and fluorescein angiography before receiving PRP. IOP was measured using a non-contact tonometer (CT-80 or CT-1P; Topcon Inc., Tokyo, Japan), and fundus photographs were taken using a 45° digital fundus camera (CR6-45NW; Canon Inc., Utsunomiya, Japan or TRC-NW8, Topcon Inc., Tokyo, Japan). OCT imaging was performed using the swept-source mode of a high-definition OCT system (DRI OCT Triton, Topcon, Tokyo, Japan). An ultra-wide-field scanning laser ophthalmoscope (Optos Optomap Panoramic 200MA; Optos PLC, Dunfermline, Scotland) allows wide-angle retinal imaging. Duration of follow-up of ocular findings is defined as the period from the initiation of PRP to the last follow-up. During follow-up periods, we checked occurrence of NVG, types and number of intravitreal injection, occurrence of vitreous hemorrhage, tractional retinal detachment, and implementation of pars plana vitrectomy. The presence of any type of glaucoma, POAG, normal-tension glaucoma NTG, NVG, and others was also investigated.

### Evaluation and management of diabetic retinopathy

The stage of DR was determined by comparison with standard photographs from the ETDRS^31^. If PDR or progression of severe NPDR was suspected in the fundus photography, fluorescein angiography was conducted. Indications of PRP were defined as PDR, very severe NPDR^31^, or aggravation of severe NPDR. Intravitreal injections of anti-vascular endothelial growth factor were given in cases of diabetic macular edema with central macular thickness of above 300 µm or vitreous hemorrhage.

### Panretinal photocoagulation

Two experienced retinal specialists (S.K, I.W.P) performed PRP according to the ETDRS and DRS.

According to DRS protocol using a standard argon-type laser PRP, the recommended settings include burns that range approximately 400 μm in size, pulse durations of 100 ms, and 200 mW of power. Laser burns (1200 to 1600) are evenly beamed or scattered on the retina away from the macula, almost to the equator. Burns were spaced at a one-burn spacing pattern. PRP was performed across 4 treatment sessions, 1 session performed per week^7^.

### Definition of DR progression

In this study, DR worsening was assessed in patients with prior PRP using the previously described composite end point of time to new proliferative event^32–34^. This composite end point takes into account clinical outcomes associated with DR worsening as defined by progression to PDR, any occurrence of newly diagnosed iris or retinal neovascularization, treatment with PRP or vitrectomy for DR-related reasons, or new cases of PDR identified by ophthalmoscopy^32–34^. The clinical experiences of patients who underwent on-study PRP were assessed by determining the incidence and timing of first on-study occurrences of vitrectomy, retinal neovascularization, or iris neovascularization. Progressive DR changes were confirmed and agreed on by the same two experienced specialists (S.K, I.W.P), each of whom was masked to the subject’s identity and to all other test results.

### Progression group and non-progression group

The patients were subdivided into progression group and non-progression group according to progression of DR: the progression group that consisted of eyes exhibiting DR progression (progression to PDR or newly developed NVI or NVE or NVG, or implementation of vitrectomy), and the ‘non-progression’ group that consisted of eyes exhibiting stationary DR.

### Statistical analysis

The baseline demographics and clinical variables were summarized by means and standard deviations or frequencies and percentages, as appropriate. The clinical characteristics of the progression group versus non-progression group were compared using unpaired t-tests or Mann–Whitney U tests for continuous values and the Chi-square test for categorical variables. Univariate and multivariate logistic regression analyses employing a forward conditional method were performed to determine the associations of various factors with progression of DR; hazard ratios (HRs) and 95% confidence intervals (CI) were reported. To avoid multicollinearity, variables correlated significantly with each other were not analyzed simultaneously. Instead, the variable with the highest significance among correlated variables was chosen. If significances were similar between correlated variables, multiple analyses were conducted separately using each variable. Kaplan–Meier survival analysis was used to compare the inter-group cumulative probability of maintenance of the DR without progression, as stratified by the significant variables derived from multivariate logistic regression. All statistical analyses were performed using SPSS version 21.0 (SPSS, Chicago, IL, USA). All P-values were two-sided and considered significant when *P* < 0.05.

## Data Availability

Data supporting the findings of the current study are available from the corresponding author on reasonable request.
